# The association of HBV infection and head and neck cancer: a systematic review and meta-analysis

**DOI:** 10.1186/s12885-024-11967-7

**Published:** 2024-02-16

**Authors:** Rukeng Tan, Xinyu Zhu, Yutong Sun, Shihao Yang, Chao Peng, Xinkai Feng, Zengyu Chen, Yiliyaer Yimamu, Guiqing Liao, Le Yang

**Affiliations:** 1 Hospital of Stomatology, Sun Yat-sen University, 56th Lingyuanxi Road, 510055 Guangzhou, Guangdong China; 2 Guangdong Province Key Laboratory of Stomatology, No. 74, 2nd Zhongshan Road, 510080 Guangzhou, Guangdong China; 3 The First People’s Hospital of Kashi Area, Xinjiang Uygur Autonomous Region, No.120, Yingbin Avenue, Kashi, People’s Republic of China

**Keywords:** Hepatitis B virus, Head and neck cancer, meta-analysis, Oral cancer, Nasopharyngeal carcinoma

## Abstract

**Background:**

Hepatitis B virus (HBV) infections is an important public health problem worldwide and closely affect extrahepatic cancer. Several recent studies have investigated the relationship between HBV infection and head and neck cancer (HNC), but their findings were inconsistent.In order to address the limitations of small sample sizes, we conducted a meta-analysis to assess the association between HBV and HNC.

**Methods:**

We systematically searched PubMed, Web of Science, Embase, Scopus, Cochrane Library, and China National Knowledge Infrastructure from inception to August 2023. Original articles published as a case-control or cohort study were included. HBV infection was identified by HBsAg, HBV DNA or ICD codes. Review articles, meeting abstracts, case reports, communications, editorials and letters were excluded, as were studies in a language other than English or Chinese. According to the MOOSE guidelines, frequencies reported for all dichotomous variables were extracted by two reviewers independently. Similarly, the outcomes of OR, RR or HR, and 95% CIs after adjusting for age and gender were collected.

**Results:**

Thirteen relevant studies and 58,006 patients with HNC were included. Our analysis revealed a positive correlation between HBV and HNC (OR = 1.50; 95% CI: 1.28–1.77). After adjusting for age and gender, the similar result (OR = 1.30; 95% CI: 1.10–1.54) was obtained. Subgroup analysis further demonstrated a significant association between HBV infection and oral cancer (OR = 1.24; 95% CI: 1.05–1.47), as well as nasopharyngeal carcinoma (OR = 1.41; 95% CI: 1.26–1.58). However, due to the limited number of studies included, the statistical significance was not reached for cancer of the oropharynx (OR = 1.82; 95% CI: 0.66–5.05), hypopharynx (OR = 1.33; 95% CI: 0.88-2.00), and larynx (OR = 1.25; 95% CI: 0.69–2.24) after adjusting for age and gender. When excluding the interference of HIV/HCV, smoking and alcohol use, the final outcome (OR = 1.17; 95% CI: 1.01–1.35) got the same conclusion.

**Conclusions:**

Our study confirmed a positive relationship between HNC, specifically oral cancer and nasopharyngeal carcinoma, and HBV infection. However, further investigation is required at the molecular level to gather additional evidence in HNC.

**Supplementary Information:**

The online version contains supplementary material available at 10.1186/s12885-024-11967-7.

## Introduction

Hepatitis B virus (HBV) infection is a significant global public health concern. The prevalence of chronic HBV infection worldwide was estimated at 4.1%, with approximately 316 million individuals infected, and HBV-related diseases caused 555,000 deaths in 2019 [1]. In 2018, the number of new cancer cases attributable to infections reached 2.2 million, accounting for 13% of all cancer cases, and HBV result in 360,000 new cancer cases [2]. In China, the burden of cancer attributed to HBV is significant, with 11.7 cases per 100,000 person-years [2]. Recent studies have highlighted the close relationship between HBV and extrahepatic cancers, including gastric cancer [3], pancreatic cancer [4], colorectal cancer [5], and lymphoma [6], as HBV infection increases the risk of malignant tumor.

Head and neck cancer (HNC) had an estimated 930,000 new cases and 460,000 related deaths worldwide in 2020 [7]. Human papillomavirus infection (HPV) is a known risk factor for oropharyngeal cancer (OPC), while Epstein-Bar virus (EBV) is primarily associated with nasopharyngeal carcinoma (NPC) [2, 8]. Several studies [9–11] found that the HBV infection rate among HNC patients is over 10%, suggesting that HBV may potentially contribute to the risk of HNC. However, the prevalence of HBV varies significantly across different parts of the world, ranging from 1.1% in Japan [12] to over 15% in China [9–11]. This variation could be attributed to regional differences in HBV prevalence. Previous studies [9–22] investigating the association between HBV and HNC risk have generated inconsistent results, likely due to small sample sizes. To overcome this limitation, we conducted a meta-analysis to comprehensively evaluate the available evidence concerning the relationship between HBV and HNC.

## Methods

### Search strategy

This study was performed according to the preferred reporting items for Meta-analysis Of Observational Studies in Epidemiology (MOOSE) guidelines (MOOSE checklist) [23]. All literature until August 31, 2023, was searched using the following databases: PubMed, Web of Science, Embase, Scopus, Cochrane Library, and China National Knowledge Infrastructure (CNKI). The following keywords and their combinations were used in PubMed: (oral OR oropharyn* OR hypopharyn* OR laryn* OR nasopharyn* OR “salivary gland” OR “head and neck” OR extrahepatic) AND (cancer OR carcinoma OR neoplasm) AND (“hepatitis b” OR HBV). The search strategies used for other database were displayed in Supplement 1. Two reviewers (ZXY and PC) independently screened all titles and abstracts and evaluated the full texts of the literature collected by the search. A third author (TRK) settled discordances.

### Inclusion criteria and exclusion criteria

The inclusion criteria for this study encompassed the following: (1) original literature published as a case-control or cohort study; (2) HBV infection was identified by HBsAg or HBV DNA or International Classification of Diseases (ICD) codes, including ICD-9 or ICD-10. (3) articles that calculated odds ratio (OR), relative risk (RR), or hazard risk (HR), along with their corresponding 95% confidence interval (CI) between HBV and HNC, or articles from which the original data could be obtained.

The exclusion criteria for this research comprised the following: (1) review articles, meeting abstracts, case reports, communications, editorials and letters; (2) articles that contained aggregated data or duplicated data from previous publications; (3) articles that lacked a normal control group or did not report the infection rate of the normal population; (4) studies in a language other than English or Chinese.

### Data extraction and quality assessment

Articles managed by EndNote X9 (Thomson ResearchSoft, Stanford, California) and data recorded by Microsoft Excel 2013 (Microsoft Corporation, Redmond, Washington). Two reviewers (SYT and YSH) extracted data from selected articles independently. The extracted information included the following: (1) study characteristics: first author’s name, year of publication, type of study, original country, data source, year of enrolment, exclusion criteria, number of control group/cohort population, number of case group, and HBV marker; (2) patient characteristics: age, gender, and cancer site; (3) data characteristics: outcomes of OR, RR or HR, and 95% CIs. Disagreements were resolved through consultation with a senior author (YL). The quality of each observational study was evaluated by two independent reviewers (CZY and FXK) using the Newcastle–Ottawa Quality Assessment Scale (NOS) [24], which included the methodological domains: selection populations, comparability of groups, and outcome of interest. The NOS scale contains 8 questions, and the highest possible score is 9 stars; Studies with a total star of 6 or less are considered low quality, whereas stars of studies with 7 or more are considered high quality.

### Statistical analysis

Statistical analysis was performed with Review Managers (version 5.4, Cochrane Collaboration, Oxford, UK) and Stata (version 12.0, Stata Corporation, College Station, Texas). The log OR takes the natural logarithm of each value of OR in the articles. The standard error of OR was derived from the log CIs, or estimated from the observed/expected number of cases for cohort studies. Since the incidence rate of cancer is low [18–20, 22] (< 0.3%), RR and HR can be approximately considered as equivalent to the OR. *I*^*2*^ and chi-square tests were applied to evaluate the statistical heterogeneity among studies, and *I*^*2*^ ≥ 50% or *p* < 0.05 indicated heterogeneity across studies. If *I*^*2*^ < 50% or *p* ≥ 0.05, a random-effects model was used to estimate the pooled ORs. Otherwise, a fixed effect model was selected. Sensitivity analysis was performed by omitting each study at a time, to verify the stability of the results of the meta-analysis when heterogeneity was apparent (*p* < 0.05). Egger’s linear regression, Begg’s rank correlation, and funnel plots were performed to test for evidence of publication bias. This study was registered with PROSPERO, CRD42023457956.

## Result

### Search results

**Fig. 1:**
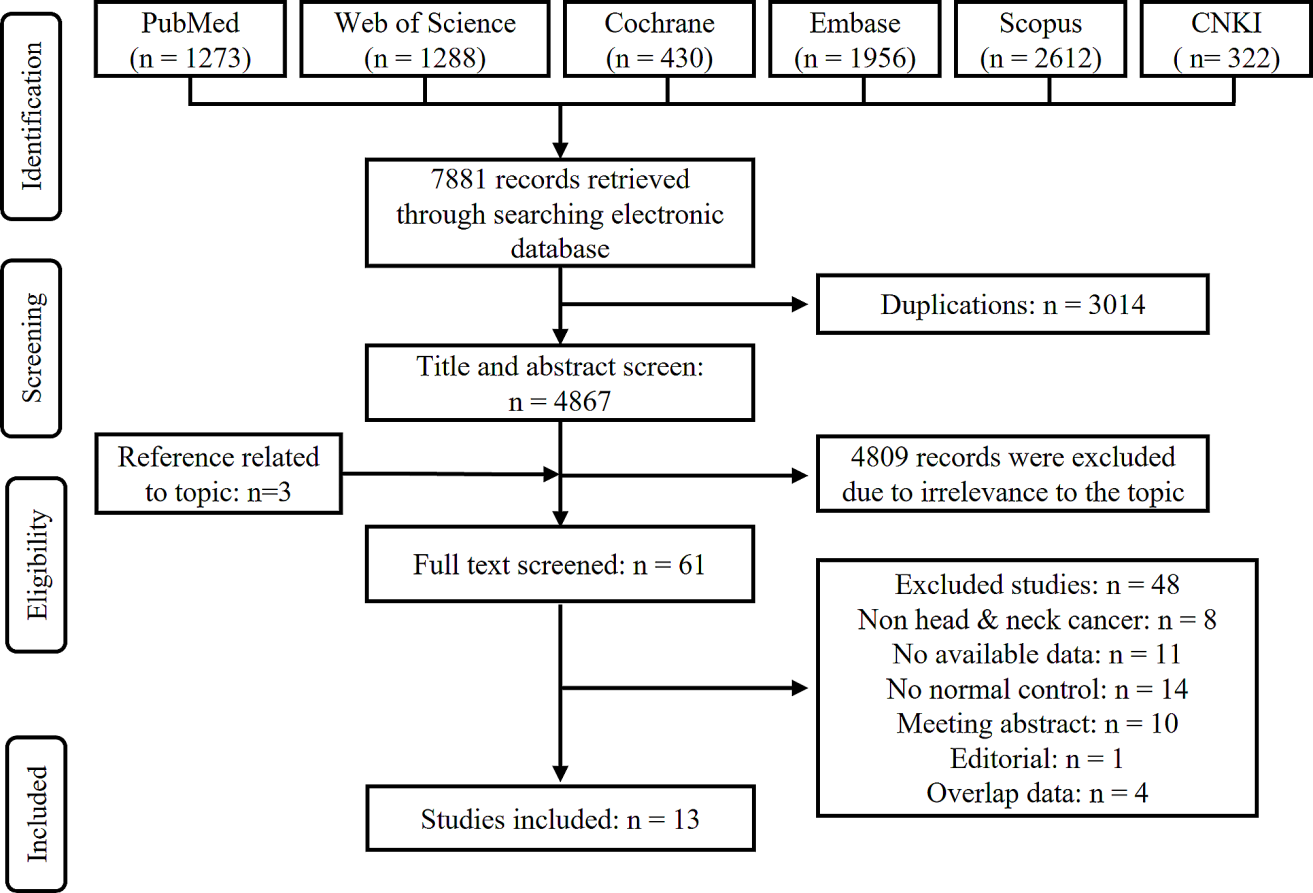
Flowchart of the literature search and article selection

The articles search obtained 7881 records from the electronic database: 1273 from PubMed, 1288 from Web of Science, 430 from the Cochrane Library, 1956 from Embase, 2612 from Scopus, and 322 from CNKI, with 4867 remaining after deduplication. After screening the titles and abstracts, 61 studies were probably eligible for inclusion. Subsequently, we read the full text of the remaining articles, and 13 independent studies were included in this study. Our workflow for the selection of relevant articles was shown in Fig. 1.

### Study characteristics

Among the included studies, there were a total of 10 case-control studies and 3 cohort studies. These studies were published between 2002 and 2020 and involved 58,006 patients with HNC. Out of the 13 studies, 7 originated from China (Guangzhou, Nanjing, Taiwan) and the remaining 6 were from non-China countries (United States, Italy, Japan, South Korea, Turkey). The primary tumor sites of HNC included the oral cavity, nasopharynx, oropharynx, hypopharynx, larynx, and salivary glands. In 10 studies, chronic HBV infections were identified by HBsAg, while the rest of the studies used ICD-9 or ICD-10 codes (Table 1). The NOS scores and characteristics of the included research were displayed in Supplement 2. It should be noted that 2 cohort studies were obtained from the same data source between 2001 and 2005. One study focused on HNC, while the other studied oral cancer. Therefore, the data from Kamiza [19] was used to estimate the overall OR, and the data from Su [18] was used for subgroup analysis.

**Table 1:** Characteristics of the included studies

First Author, Year	Country	Year of enrolment	Control group/ Cohort population	Event of control group	Number of control group	Cancer site	Event of case group	Number of case group	HBV marker
**Case-control studies (** ***n*** ** = 10)**									
Takata, 2002 [12]	Kitakyushu, Japan	1989–1998	impacted teeth	3	350	442 oral cavity	5	442	HBsAg
Ye, 2015 [9]	Guangzhou, China	2008/1-2013/5	healthy population	97	680	711 nasopharynx	112	711	HBsAg
Wei, 2017 [10]	Guangzhou, China	2008–2014	cancer free patients	723	5715	4152 nasopharynx	791	4152	HBsAg
Kocoglu, 2018 [13]	Istanbul, Turkey	2000–2014	healthy volunteers	3168	96,000	204 head and neck	12	204	HBsAg
An, 2018 [21]	Seoul, South Korea	2007–2014	cancer-free controls	4401	118,891	1750 head and neck	95	1750	HBsAg
Lu, 2018 [11]	Guangzhou, China	2007–2016	residents in South China	14,823	169,211	3323 nasopharynx	535	3323	HbsAg
Mahale, 2019 [17]	Maryland, USA	1993–2013	cancer-free population	966	200,000	4417 salivary glands/14,479 oral cavity/8515 oropharynx/12,334 larynx/4141 other head and neck sites(1165 nasopharynx/2251 hypopharynx)	-	-	ICD-9
Donà, 2019 [14]	Treviso, Italy	2000–2018	cancer free patients	26	1518	107 oral cavity/202 oropharynx/83 hypopharynx/382 larynx	35	774	HBsAg
Komori, 2020 [15]	Okayama, Japan	2008–2017	non-HNC patient	5	495	512 head and neck(152 oral cavity/10 nasopharynx/57 oropharynx/120 hypopharynx/80 larynx/26 salivary glands/67 others)	5	512	HBsAg + HBcAb
Tian, 2020 [16]	Nanjing, China	2008–2016	cancer-free subjects	625	11,361	378 oral cavity/82 nasopharynx/82 hypopharynx/345 larynx	80	887	HBV sero-markers
**Cohort studies**									
Kamiza, 2016 [19]	Taiwan, China	2000–2005	healthy population	233	63,552	47 head and neck	47	12,369	ICD-9-CM
Song, 2019 [20]	Nanjing, China	2004/6-2008/7	healthy population	395	481,377	20 oral cavity	20	15,355	HBsAg
Hong, 2020 [22]	Seoul, South Korea	2003–2013	no HBV or HCV population	638	500,680	32 head and neck(3 larynx)	32	26,665	ICD-10

### Correlation between HBV and HNC

**Fig. 2:**
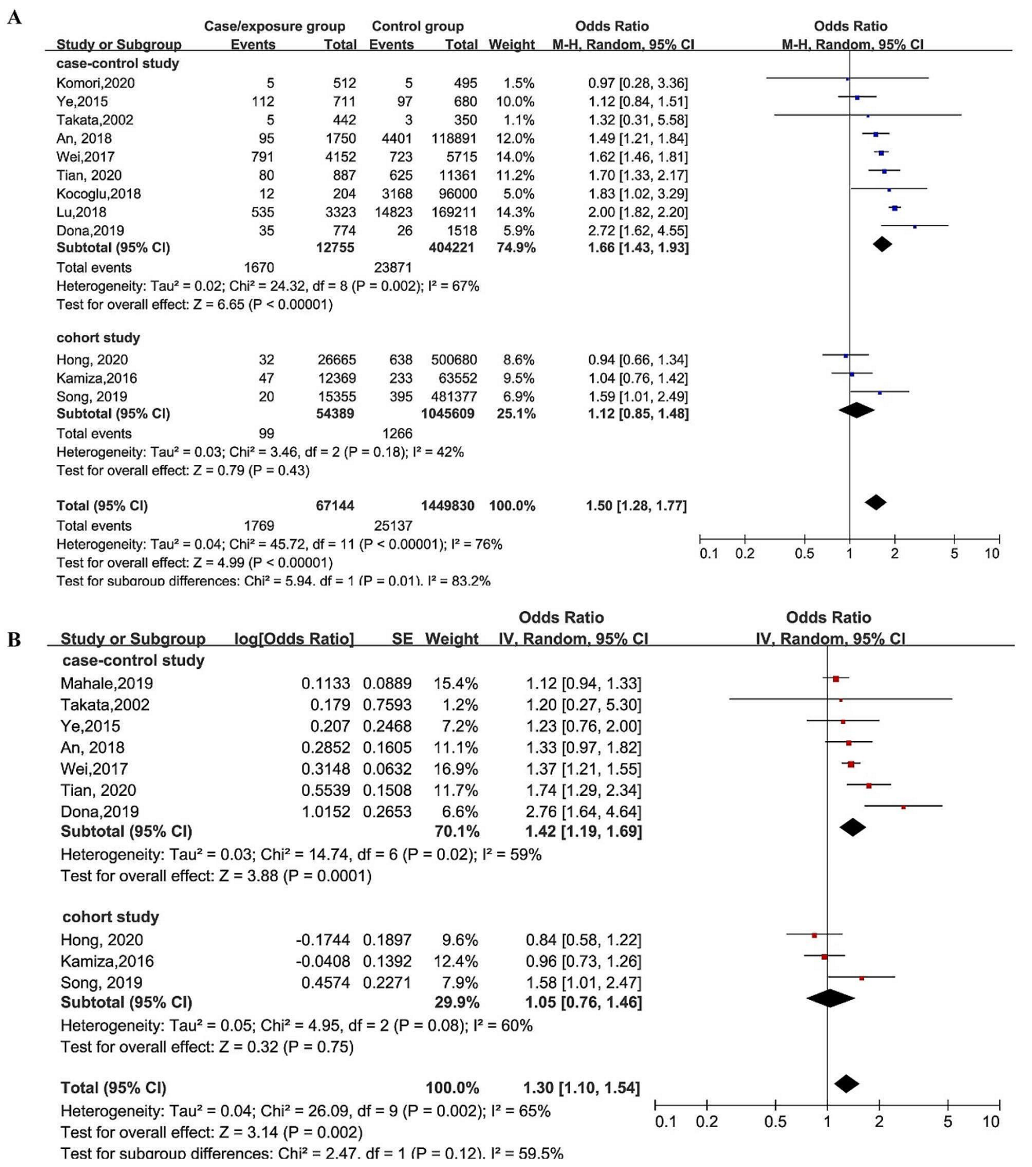
Forest plot of the overall outcome, with unadjusted data (A) and with data adjusted for age and gender (B), for the association between HBV and HNC

As shown in Fig. 2, twelve studies have investigated the association between HBV and the risk of HNC, with a pooled OR of 1.50 (95% CI: 1.28–1.77, *p* = 0.01), and statistically significant heterogeneity (*I*^*2*^ = 76%, *p* < 0.00001) was found across studies. The symmetrical funnel plot (Supplement Fig. 1A) and Begg’s test (*p* = 0.837) and Egger’s test (*p* = 0.129) showed no significant evidence of publication bias. Furthermore, sensitivity analysis was performed and showed that no individual study could influence the pooled OR estimate and the results were reliable and robust (Supplement Fig. 1B). After adjusting for age and gender, ten studies were included with a pooled OR of 1.30 (95% CI: 1.10–1.54, *p* = 0.002) and less heterogeneity (*I*^*2*^ = 65%, *p* = 0.002). No significant evidence of publication bias was found and the result was robust (Supplement Fig. 1 C-D). The results of cohort studies did not show statistical differences. However, both the overall studies and case-control studies, with or without adjustment, confirmed a significant association between HBV infection and HNC.

### Subgroup analysis

**Fig. 3:**
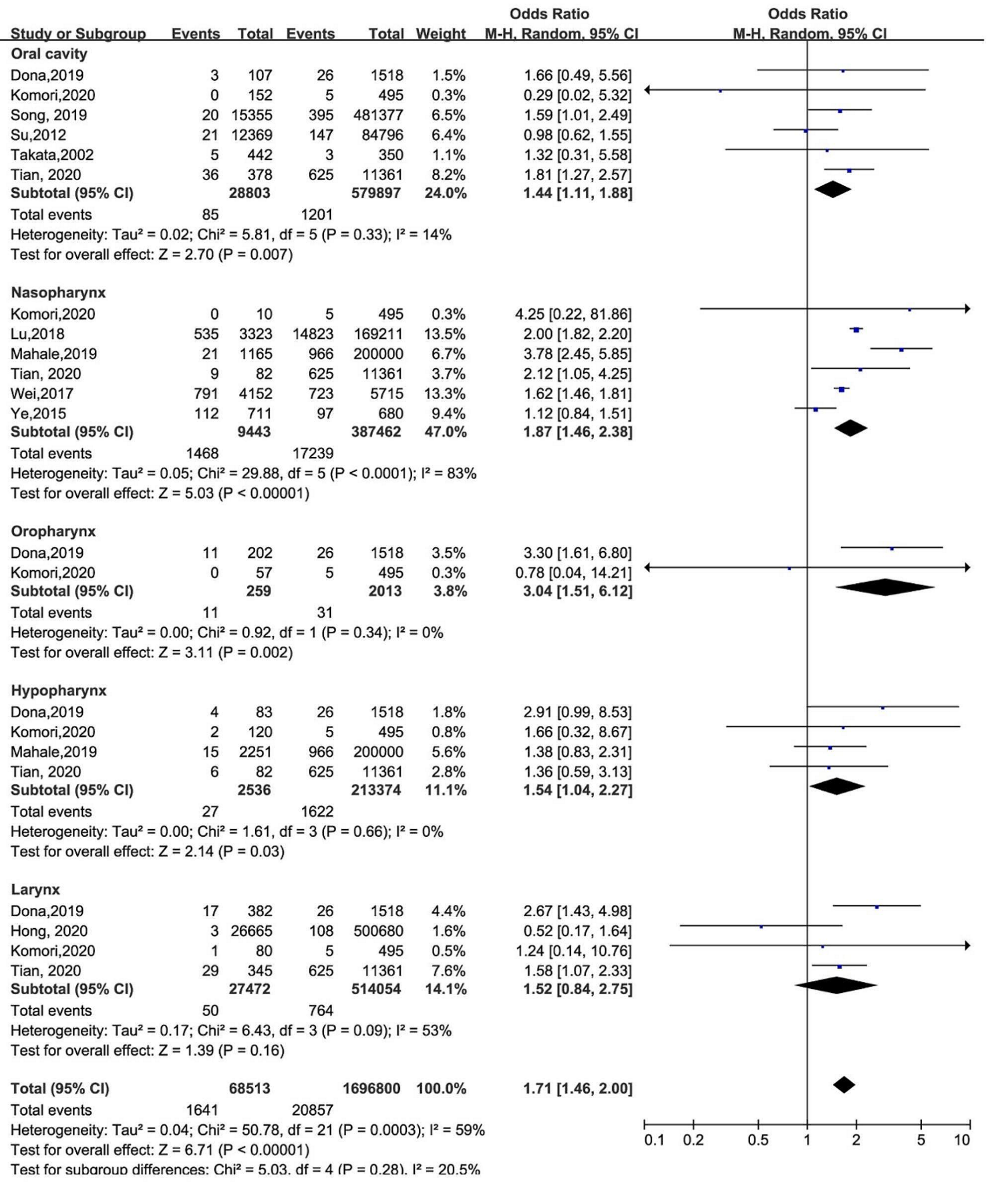
Forest plot for the association between HBV and different sites of HNC

According to the primary tumor site, we performed a subgroup analysis to investigate the relation between HBV and oral cancer, nasopharyngeal carcinoma, oropharyngeal cancer, hypopharyngeal cancer, laryngeal cancer. The results of subgroup analysis with unadjusted data were as follow (Fig. 3). Six studies have examined the relationship between HBV and oral cancer, and the pooled OR was found to be 1.44(95% CI: 1.11–1.88; *p =* 0.007; random effects; *I*^*2*^ = 14%; *p =* 0.33). Similarly, six studies investigated the association between HBV and the risk of nasopharyngeal carcinoma, and the pooled OR was 1.87(95% CI: 1.46–2.38; *p* < 0.00001; random effects; *I*^*2*^ = 83%; *p* < 0.0001). Furthermore, sensitivity analysis showed that no individual study had an undue influence, and the OR remained consistent throughout (Supplement Fig. 1F). Additionally, significant statistically pooled OR emerged for oropharyngeal cancer (OR = 3.04; 95% CI: 1.51–6.12; *p* = 0.002; random effects; *I*^*2*^ = 0%, *p* = 0.34), hypopharyngeal cancer (OR = 1.54; 95% CI: 1.04–2.27; *p* = 0.03; random effects; *I*^*2*^ = 0%, *p* = 0.66). Nevertheless, the result of laryngeal cancer (OR = 1.52; 95% CI: 0.84–2.75; *p* = 0.16; random effects; *I*^*2*^ = 53%, *p* = 0.09) did not have statistical differences. To reduce confounding factors, we included 5 studies without HIV or HCV patients (Table 2), and the outcomes of OR was 1.46(95% CI: 1.13, 1.88; *p* = 0.004; random effects; *I*^*2*^ = 86%, *p* < 0.00001).

**Table 2:** Results of meta-analysis for subgroup with or without adjustment

Subgroup analysis	Studies, no.	Analysis model	Pooled OR (95% CI)	*p* value^*^	Study Heterogeneity				Egger’s test	Begg’s test
					χ^2^	dƒ	I^2^, %	*p* value^*^		
**Before adjustment**										
Excluded HIV and HCV	5	M-H, Random	1.46(1.13, 1.88)	**0.004**	29.50	4	86	**< 0.00001**	**0.019**	0.086
**After adjustment for age and gender**										
Cancer site	19	IV, Random	1.39(1.19, 1.62)	**< 0.0001**	42.64	18	58	**0.0009**	0.333	0.441
Oral cavity	6	IV, Fixed	1.24(1.05, 1.47)	**0.01**	9.30	5	46	0.10	0.461	1.000
Nasopharynx	4	IV, Fixed	1.41(1.26, 1.58)	**< 0.0001**	4.30	3	30	0.23	0.382	0.734
Oropharynx	2	IV, Random	1.82(0.66, 5.05)	0.25	6.79	1	85	**0.009**	/	/
Hypopharynx	3	IV, Fixed	1.33(0.88, 2.00)	0.17	1.97	2	0	0.37	/	/
Larynx	4	IV, Random	1.25(0.69, 2.24)	0.46	16.46	3	82	**0.0009**	/	/
Excluded HIV and HCV	5	IV, Random	1.24(1.01, 1.51)	**0.04**	9.66	4	59	0.05	0.363	0.462
**After adjustment for age, gender, alcohol use and smoking**										
Head and neck cancer	6	IV, Fixed	1.26(1.15, 1.38)	**< 0.00001**	9.17	5	45	0.10	0.654	0.721
Excluded HIV and HCV	3	IV, Fixed	1.17(1.01, 1.35)	**0.03**	0.92	2	0	0.63	/	/

M-H = Mantel-Haenszel; IV = inverse variance; OR = odds ratio; CI = confidence interval; dƒ = degree of freedom;

^*^Statistical significant results are shown in bold

To eliminate the influence of age and gender, we performed the meta-analysis with adjusted data (Table 2). The robust outcomes showed a significant association between HBV infection and oral cancer (OR = 1.24; 95% CI: 1.05–1.47; *p* = 0.01; fixed effect; *I*^*2*^ = 46%, *p* = 0.10), as well as nasopharyngeal carcinoma (OR = 1.41; 95% CI: 1.26–1.58; *p* < 0.0001; fixed effect; *I*^*2*^ = 30%, *p* = 0.23). The results of Begg’s test, and Egger’s test showed no significant evidence of publication bias. However, relatively few studies investigate the association between HBV and oropharyngeal cancer, hypopharyngeal cancer, laryngeal cancer. And no significant statistically pooled OR emerged for oropharyngeal cancer (OR = 1.82; 95% CI: 0.66–5.05; *p* = 0.25; random effects; *I*^*2*^ = 85%, *p* = 0.009), hypopharyngeal cancer (OR = 1.33; 95% CI: 0.88-2.00; *p* = 0.17; fixed effect; *I*^*2*^ = 0%, *p* = 0.37), laryngeal cancer (OR = 1.25; 95% CI: 0.69–2.24; *p* = 0.46; random effects; *I*^*2*^ = 82%, *p* = 0.0009), but only 2–4 studies were included in this analysis. Similarly, the subgroup analysis of five studies without HIV or HCV patients was performed (OR = 1.24; 95% CI: 1.01–1.51; *p* = 0.17; random effects; *I*^*2*^ = 59%, *p* = 0.05) and no significant evidence of publication bias was found.

After further adjusting for smoking and alcohol use (Table 2), it was observed that HBV infection had a clear connection with HNC (OR = 1.26; 95% CI: 1.15–1.38; *p* < 0.00001; fixed effect; *I*^*2*^ = 45%, *p* = 0.10), considering the significant role played by smoking and alcohol use in the occurrence of HNC. The final outcome (OR = 1.17; 95% CI: 1.01–1.35; *p* = 0.03; fixed effect; *I*^*2*^ = 0%, *p* = 0.63), which included three studies without HIV or HCV patients, further supported the strong association between HBV and HNC.

## Discussion

Overall, our meta-analysis displayed a robust relation between HBV and HNC risk, and significant heterogeneity was observed. But, understandably, HNC in different locations exhibits considerable heterogeneity in biological behavior and therapeutic response. Therefore, we further conducted subgroup analyses by primary tumor site to explore the association. Then we found NPC had a relatively strong connection with HBV, while OC had a weaker but still significant association with HBV. Heterogeneity across studies was low for all subgroups but nasopharynx, oropharynx, and larynx. Excluding the interference of HIV/HCV, age, and gender, the final outcome still confirmed the closed association between HBV and HNC.

HBV, a DNA virus, has partly double-stranded circular DNA and has been linked to the development of hepatocellular carcinoma in depth [25]. Recently, increasing studies have focused on the connection between HBV and extrahepatic cancer, and molecular pathways in carcinogenesis [26]. Emerging evidence shows that HBV DNA copies were detected in several extrahepatic cancer tissues, such as gastric cancer, pancreatic cancer [10, 27], which suggested that HBV infection probably increased the risk of extrahepatic cancer by DNA repair gene mutations. A Mendelian randomization study confirmed a cause-and-effect relationship between chronic HBV infection and cervical cancer, gastric cancer by analyzing millions of single-nucleotide polymorphisms [28]. And several articles reported that hepatitis B virus X (HBx) protein expressed in gastric cancer, and pancreatic cancer [20, 29], especially, HBx protein was found to promote pancreatic ductal adenocarcinoma through PI3K/AKT signaling pathway [29]. Long-term chronic HBV infections profoundly affect the tumor microenvironment and immune system [30, 31], non-small cell lung cancer patients with HBV infection had more PD-L1 expression on immune cells and longer OS and PFS than patients without chronic hepatitis B infection [32, 33]. Moreover, several HBV-induced cancer-related signaling pathways in hepatocellular carcinoma were reported in extrahepatic tissue carcinogenesis as follows: (1) Wnt pathway [34]; (2) PI3K/Akt signaling pathway [29, 35]; (3) METTL3-mediated MYC mRNA m6A modification [36].

There were a few lines of evidence to support the association between chronic HBV infection and HNC, especially in NPC. Xie et al. detected the HBx protein and mRNA in adenoid cystic carcinoma and Warthin’s tumor, and HBx was expressed in both the cytoplasm and nucleus [37]. After analysis of 1301 patients with NPC in China, HBV infection was confirmed as an independent risk factor in patients with advanced NPC [38]. Weng et al. [39] reported that patients with early-stage NPC had worse 5-year overall survival, disease-free survival, relapse-free survival, and distant metastasis-free survival rather than those patients in HBsAg(−) group, and antiviral therapy might lead to a better prognosis for the HBV-infected NPC patients. The incidence of HBV reactivation and HBV-related hepatitis was 9.1% and 2.5%, respectively, when NPC patients received immunosuppressive therapies [40]. Furthermore, Huang et al. found that HBx protein could regulate yes-associated protein 1 to promote NPC invasiveness through EMT [41].

As chemotherapy, radiotherapy, target therapy, and immunotherapy are widely used as part of the comprehensive treatment for head and neck cancer, HBV reactivation is regarded as a key problem when the immunosuppressive effect of the administered treatment, which leads to liver damage to interrupt the anti-cancer therapy [26, 40]. And a meta-analysis reported that the risk for HBV reactivation of HNC without antiviral prophylaxis arrived at 27.5% [42]. A retrospective study found that patients with HNC who had HBV infection had worse overall survival and progression-free survival compared to HNC patients without HBV infection. Interestingly, the study did not find any association between HBV infection and the TNM stage or grade of HNC [43].

According to our knowledge, this is the first article to systematically analyze the association between HBV and HNC risk. Nevertheless, this study also had several limitations which deserve discussion. Firstly, significant risk factors like tobacco smoking, alcohol drinking, and viral status (HPV, EBV) are known to be related to HNC and its prognosis. Unfortunately, most studies lack this detailed individual information, which might result in a lack of control for potential confounding factors. Secondly, the small sample sizes and the variation in hepatitis B and cancer prevalence across different areas could contribute to the heterogeneity observed, as the high incidence of hepatitis B and HNC in developing countries, but relatively low in developed countries [1, 7, 44, 45]. Therefore, further research requires epidemiological evidence with larger sample sizes and higher-quality functional studies. Lastly, as for the selection of control group population, most studies collected hospital patients, which might introduce some different characteristics related to HBV exposures than the normal population. As a result, there was inevitably a selection bias that may lead to evaluation bias of the relationship between HBV and HNC.

In conclusion, our study confirmed the positive relation between HNC, especially OC and NPC, and HBV. What is more important, further strong epidemiological evidence and more biological mechanism study were needed to investigate the association between HBV and HNC. Meanwhile, the need for further research to determine the value of HBV screening and prophylaxis for patients with HNC before receiving immunosuppressive therapies.

### Electronic supplementary material

Below is the link to the electronic supplementary material.

**Supplement 2 data extraction**

**Supplementary Material 2**: **Supplement Figure 1**. The funnel plots and sensitive analysis for HNC before adjustment (A,B) and after adjustment (C,D) and nasopharyngeal carcinoma before adjustment (E,F). The sensitive analysis was performed when *p* < 0.05 in heterogeneity

**Supplementary Material 3**: **Supplement Figure 2**. The funnel plots and sensitive analysis for cancer sites before adjustment (A,B) and after adjustment (E,F). Although publication bias was found in the analysis for excluding HIV and HCV patients before adjustment for age and gender, the sensitivity analysis showed the result was robust (C,D). The sensitive analysis was performed when *p* < 0.05 in heterogeneity

**Supplementary Material 4**: **Supplement Figure 3**. The funnel plots and sensitive analysis for head and neck cancer after adjusting for age, gender, alcohol use and smoking (A,B). The funnel plot for head and neck cancer after excluding HIV and HCV patients and adjusting for age, gender, alcohol use and smoking (C)

**Supplement 1 Search strategy**

## Data Availability

Data were taken from publicly available publications and as such can be widely accessed. All data generated or analyzed during this study are included in this published article [and its supplementary information files]. The data used and/or analyzed during the current study are available from the corresponding author on reasonable request.

## Abbreviations

HBV: Hepatitis B virus
HNC: Head and neck cancer
OPC: Oropharyngeal cancer
NPC: Nasopharyngeal carcinoma
MOOSE: Meta-analysis Of Observational Studies in Epidemiology
CNKI: China National Knowledge Infrastructure
ICD: International Classification of Diseases
OR: Odds ratio
RR: Relative risk
HR: Hazard risk
CI: 95% confidence interval
NOS: Newcastle–Ottawa Quality Assessment Scale
HBx: Hepatitis B virus X
