# Supplementary figures and images for: The association of HBV infection and head and neck cancer: a systematic review and meta-analysis
Source: BMC Cancer. 2024 Feb 16;24:225. doi: 10.1186/s12885-024-11967-7 (PMC10874002; doi:10.1186/s12885-024-11967-7)

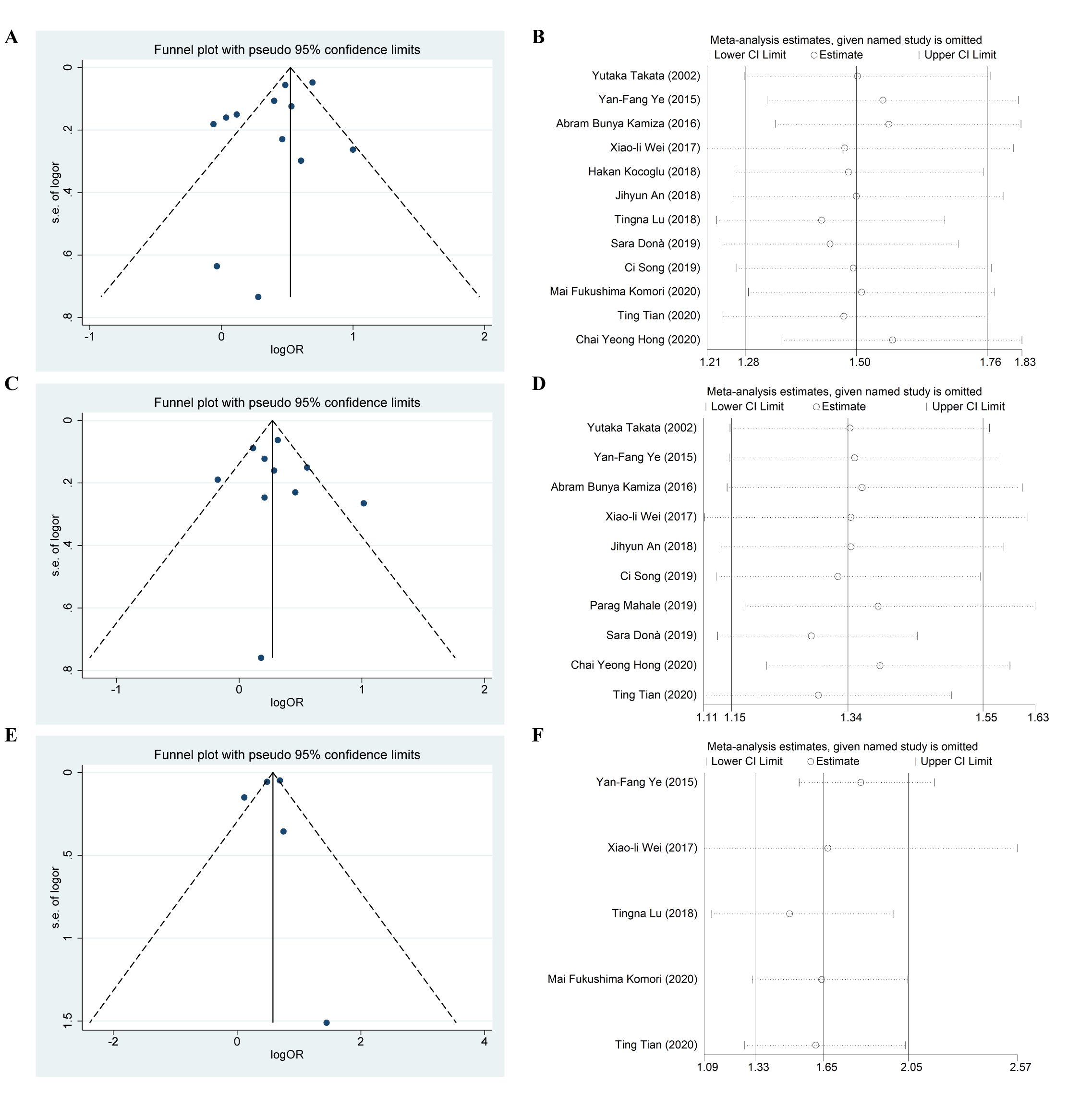

Supplement: Supplementary file 2 — Supplementary Material 2: Supplement Figure 1. The funnel plots and sensitive analysis for HNC before adjustment (A,B) and after adjustment (C,D) and nasopharyngeal carcinoma before adjustment (E,F). The sensitive analysis was performed when p < 0.05 in heterogeneity [file 12885_2024_11967_MOESM2_ESM.tif]

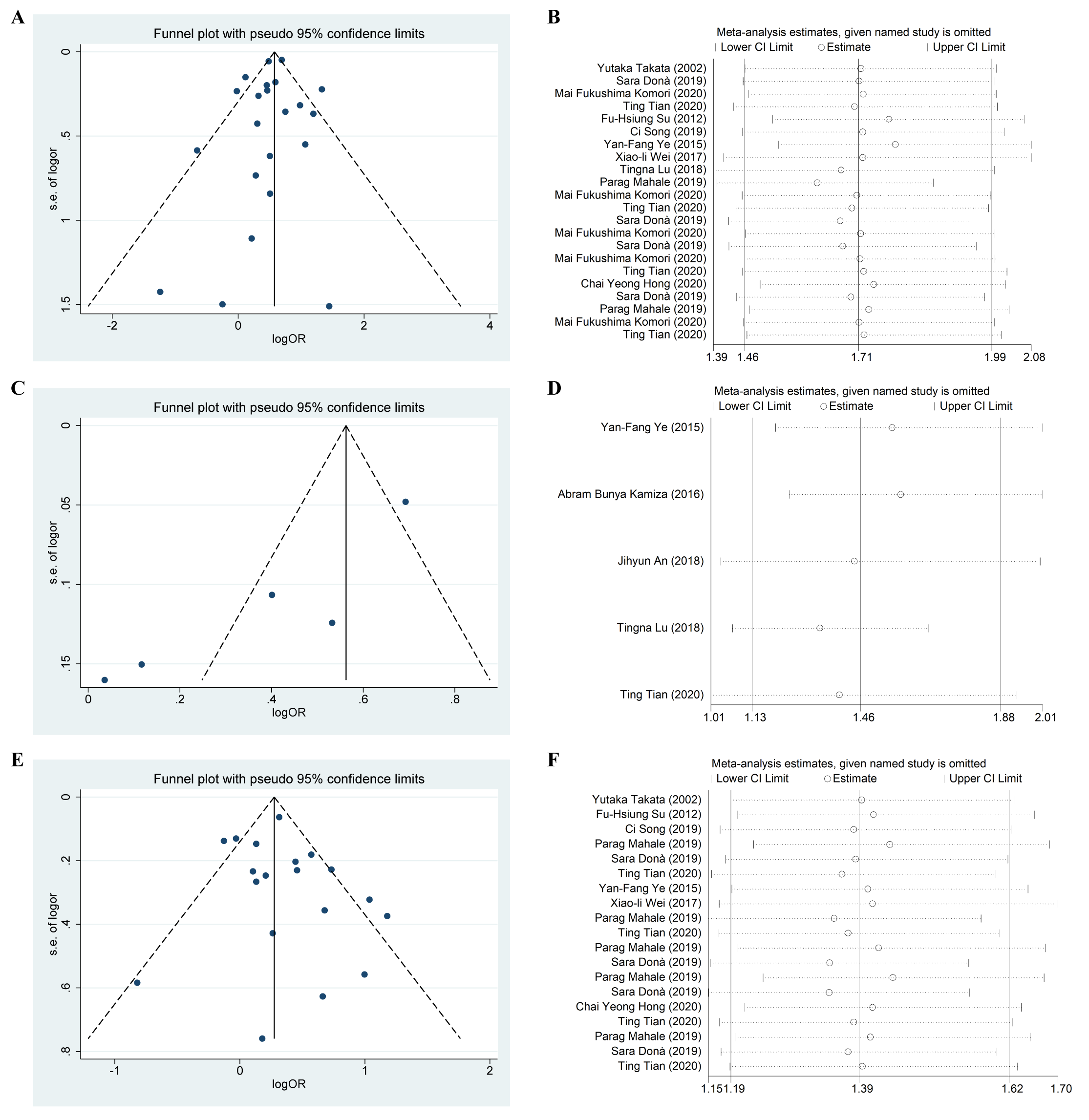

Supplement: Supplementary file 3 — Supplementary Material 3: Supplement Figure 2. The funnel plots and sensitive analysis for cancer sites before adjustment (A,B) and after adjustment (E,F). Although publication bias was found in the analysis for excluding HIV and HCV patients before adjustment for age and gender, the sensitivity analysis showed the result was robust (C,D). The sensitive analysis was performed when p < 0.05 in heterogeneity [file 12885_2024_11967_MOESM3_ESM.tif]

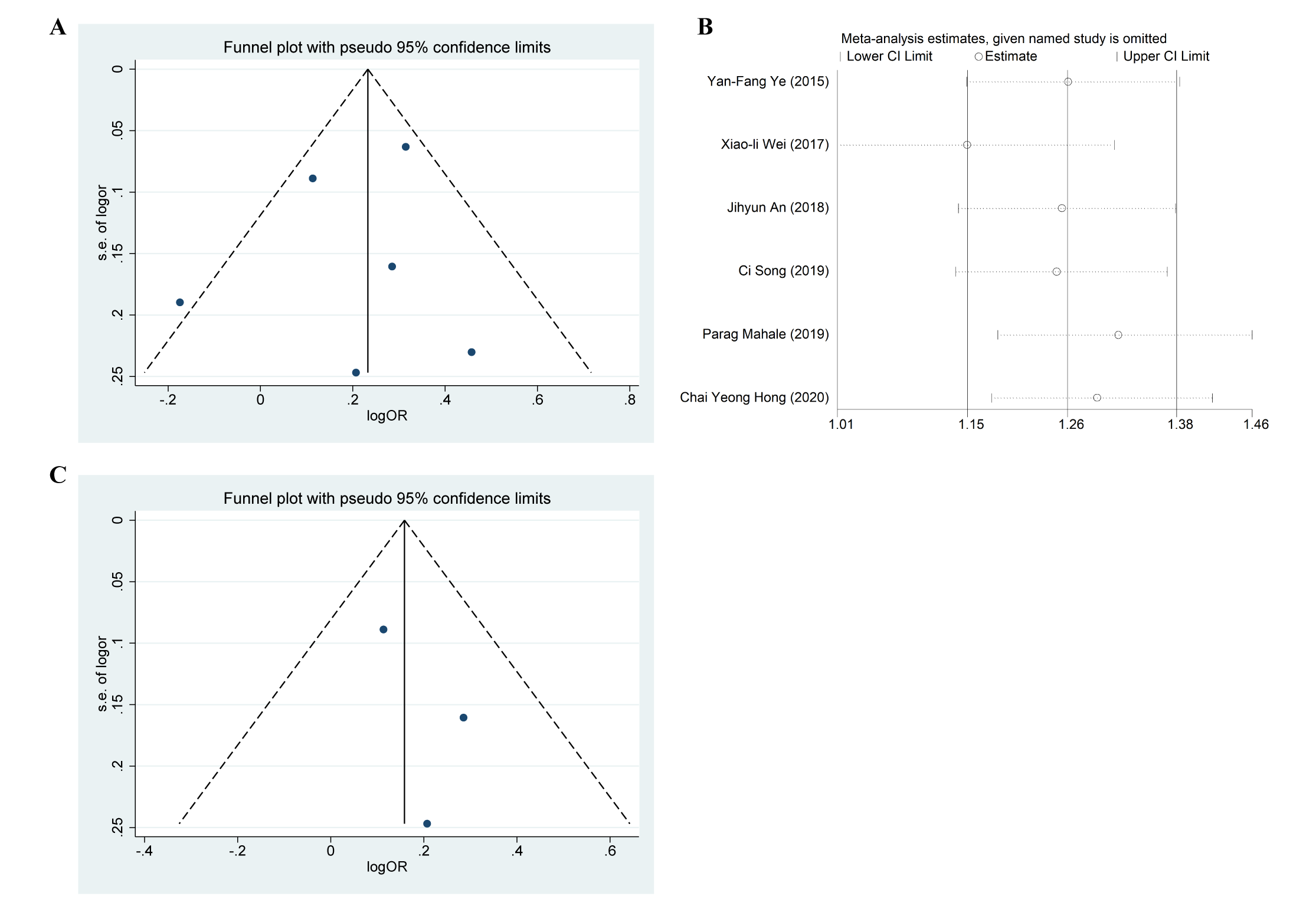

Supplement: Supplementary file 4 — Supplementary Material 4: Supplement Figure 3. The funnel plots and sensitive analysis for head and neck cancer after adjusting for age, gender, alcohol use and smoking (A,B). The funnel plot for head and neck cancer after excluding HIV and HCV patients and adjusting for age, gender, alcohol use and smoking (C) [file 12885_2024_11967_MOESM4_ESM.tif]
