# Supplementary material for: The association of HBV infection and head and neck cancer: a systematic review and meta-analysis
Source: BMC Cancer. 2024 Feb 16;24:225. doi: 10.1186/s12885-024-11967-7 (PMC10874002; doi:10.1186/s12885-024-11967-7)
Supplement: Supplementary file 5 — Supplement 1 Search strategy [file 12885_2024_11967_MOESM5_ESM.docx]

# Search strategy

PUBMED

(oral OR oropharyn* OR hypopharyn* OR laryn* OR nasopharyn* OR "salivary gland" OR "head and neck" OR extrahepatic) AND (cancer* OR carcinoma* OR neoplasm*) AND ("hepatitis b" OR HBV)

WOS

((ALL=(oral OR oropharyn* OR hypopharyn* OR laryn* OR nasopharyn* OR "salivary gland" OR "head and neck" OR extrahepatic)) AND ALL=(cancer OR carcinoma OR neoplasm)) AND ALL=("hepatitis b" OR HBV)

Cochrane

(oral OR oropharyn* OR hypopharyn* OR laryn* OR nasopharyn* OR "salivary gland" OR "head and neck" OR extrahepatic):ti,ab,kw AND (cancer OR carcinoma OR neoplasm):ti,ab,kw AND ("hepatitis b" OR HBV):ti,ab,kw

Scopus

TITLE-ABS((oral OR oropharyn* OR hypopharyn* OR laryn* OR nasopharyn* OR "salivary gland" OR "head and neck" OR extrahepatic) AND (cancer OR carcinoma OR neoplasm) AND ("hepatitis b" OR HBV))

Embase

(oral:ti,ab,kw OR oropharyn*:ti,ab,kw OR hypopharyn*:ti,ab,kw OR laryn*:ti,ab,kw OR nasopharyn*:ti,ab,kw OR 'salivary gland':ti,ab,kw OR 'head and neck':ti,ab,kw OR extrahepatic:ti,ab,kw) AND (cancer*:ti,ab,kw OR carcinoma*:ti,ab,kw OR neoplasm*:ti,ab,kw) AND ('hepatitis b':ti,ab,kw OR hbv:ti,ab,kw)

CNKI

(TKA= hepatitis b + HBV) AND (TKA= head and neck + oral + nasopharynx + oropharynx + larynx + extrahepatic) AND (TKA= tumor + cancer)
